# A rare case of recurrent eccrine poroma underlying gluteal abscess

**DOI:** 10.1016/j.ijscr.2020.08.042

**Published:** 2020-09-02

**Authors:** Abdulkarim Hasan, Khalid Nafie, Khaled Monazea, Alsayed Othman, Abdoh Salem, Amal Ismail

**Affiliations:** a Department of Pathology, Faculty of Medicine, Al-Azhar University, Cairo, 11884, Egypt; b Prince Mishari Bin Saud Hospital, Baljurashi, 65888, Saudi Arabia; c Department of Surgery, Faculty of Medicine, Al-Azhar University, Assiut, 71524, Egypt; d Department of Surgery, Faculty of Medicine, Al-Azhar University, Cairo, 11884, Egypt; e Department of Pharmacy Practice, Unaizah College of Pharmacy, Qassim University, Unaizah, Qassim, Saudi Arabia

**Keywords:** Eccrine poroma, Gluteal abscess, Gluteal region, Simple excision

## Abstract

**Introduction:**

Eccrine poroma is a benign adnexal neoplasm originating from epidermal sweat ducts mainly affecting the palmoplantar skin, buttock as a location of origin of poroma has rarely been reported, it is the first reported case of recurrent poroma in this site.

**Presentation of case:**

We report an unusual case of eccrine poroma in the gluteal region of a 62 years old female associated with gluteal abscess, surgically excised with histologically clear margins, then recurred after one and half year with a given history of mild trauma.

**Discussion:**

Given the uncommonness of these neoplasms, there stays a general lack of data on pathogenesis, atypical presentation, atypical sites, recurrence and risk of malignant transformation into porocarcinoma.

**Conclusion:**

Gluteal skin is an unusual location for a benign poroma. Early recognition and appropriate treatment at the initial presentation by complete resection with histopathology confirmation and follow-up are crucial to ruling out other diagnoses, such as lesions of malignant transformation.

## Highlights

• Poroma affects gluteal skin.
• Trauma is a possible cause.
• We recommend excision and pathology.

## 1 Introduction

Benign eccrine poroma (EP) was first described by Pinkus et al. in 1956 as a benign tumor originating from the intraepidermal eccrine part of a sweat gland duct [1]. Its incidence is approximately 0.001%–0.008% of all skin biopsy specimens and usually presents as a painless, solitary lesion, usually affecting the palms, the soles, or the feet [2]. EP is less commonly reported in the finger, posterior side of the hand, chest, forehead, nose, and scalp [3]. To our knowledge, two cases of poroma on the buttock region were reported in the literature but no recurrent poromas were reported at this anatomical site.

The pathogenesis is unclear, but its etiology is associated with a history of trauma, radiation exposure, viral infection, or actinic damage [3]. The typical clinical presentation is a slow-growing asymptomatic dome-shaped nodular lesion, plaque, often uncolored, or pigmented [4].

As EP is a benign adnexal neoplasm, treatment is curative, simple excision is the current treatment of choice for the deep lesions [5]. However, recurrence or more seriously malignant transformation is a possibility. This work has been reported in line with SCARE criteria [6].

## 2 Presentation of case

We report a case of a 62-year-old female presenting with clinical features of a left-side gluteal abscess. Upon physical examination, a firm nodule was palpated; the white blood cell count was 9500/μL, and a random serum glucose level was measured to 140 mg/dL. Surgical drainage and excision of the nodule were conducted. Microbiological examination based on culture and sensitivity testing of the drained fluid revealed E.coli organism susceptible to amikacin and cefepime. The resected nodule was sent to the histopathology laboratory and cefepime medical treatment was advised.

**Fig. 1:**
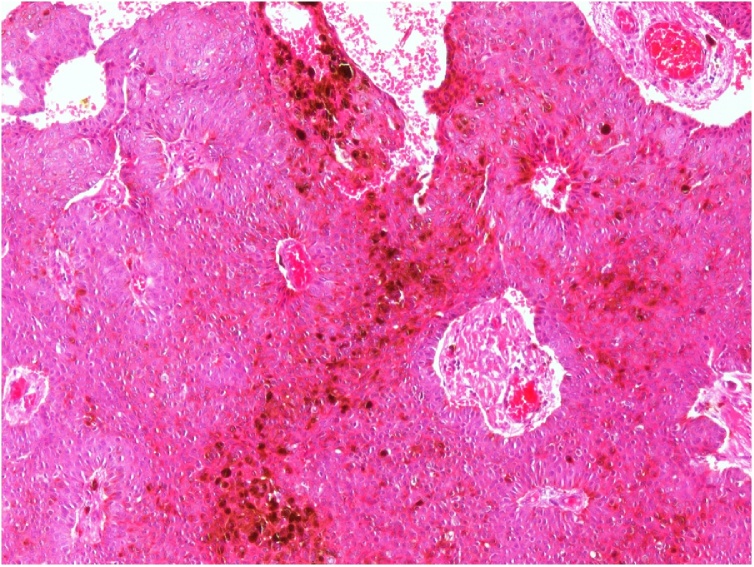
**A**: Dermal proliferation of benign-looking small uniform cells with ductal differentiation (Hematoxylin and Eosinstain, 100×). **B**: Dermal proliferation of eccrine poroma (EP) with melanin pigmentation and associated inflammatory cells (H&E stain, 100×).

Gross pathological examination revealed a fibro-fatty tissue piece measuring 6.5 × 2.5 × 1.2 cm covered by a skin piece, with an underlying, relatively circumscribed soft-to-firm yellowish nodule measuring 3.5 × 2 cm with a least surgical margin of 0.2 cm. Microscopic examination showed downward growth attached to the epidermis and reaching into the deep dermis, composed of cords and nests of small keratinocytes; the nests were sharply delimited from the adjacent epidermis; also duct-like structures and occasional islands of squamous epithelium were seen. Dermal reactive vessels and mixed inflammatory cells with neutrophil collections were present (Fig. 1A and B). The reporting pathologist suggested a diagnosis of poroma vs low grade malignant tumor; subsequently, a blinded consultation with three other pathologists confirmed the diagnosis of EP with clear margins. The patient showed a good response for the treatment with no symptoms or remnant mass lesion could be detected in the regular follow-up during the next 12 months.

**Fig. 2:**
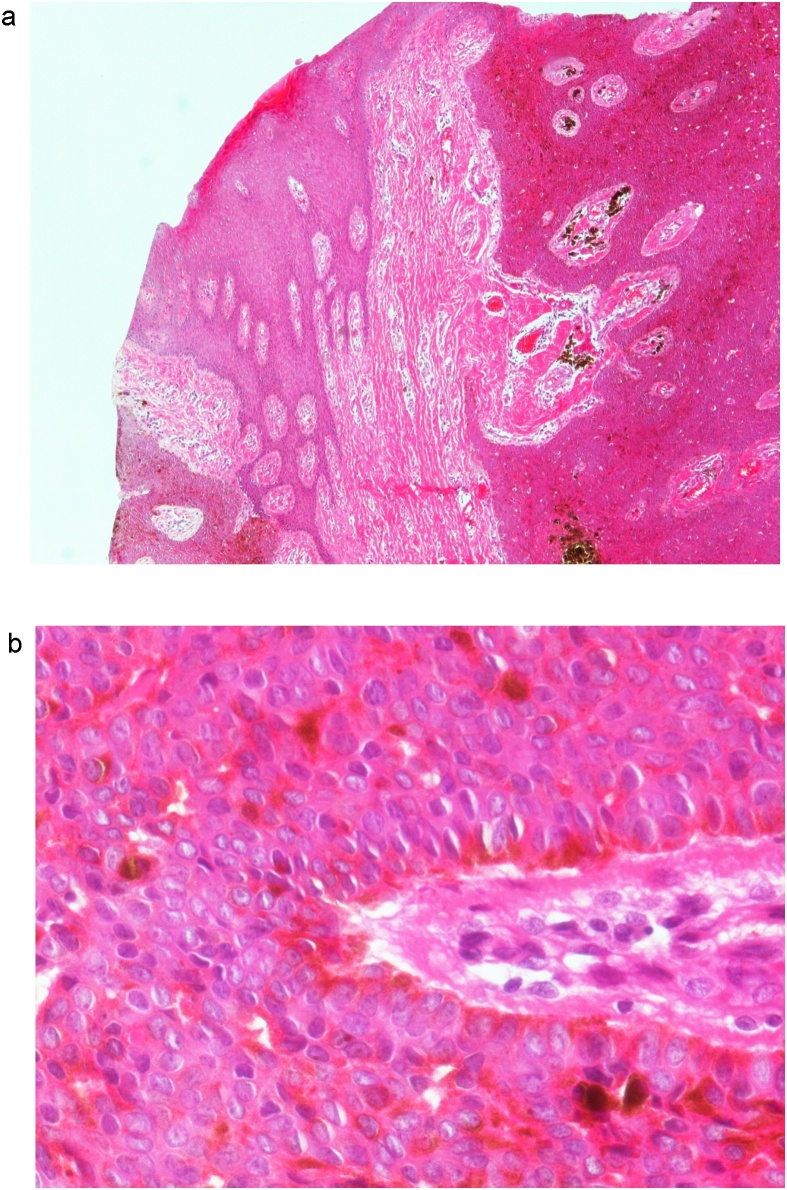
**A**: A broad anastomosing tumor extending from the epidermis to the dermis with multifocal fibrovascular cords and structures (H&E stain, 40×). **B**: On the high-power view, the tumor cells were seen to be composed of packed small uniform cells with central round-to-oval nuclei and with occasional prominent nucleoli and melanin pigment (H&E stain, 400×).

After 17 months from the operation, the patient returned with a recurrent gluteal abscess at the same site; surgical drainage was performed, and skin thickening was observed then excised. Culture and sensitivity test results revealed *Staphylococcus aureus* susceptible to amoxicillin/ clavulanic acid which was advised to the patient. The specimen received in the histopathology laboratory was partially fragmented revealing a fairly defined nodule measured 1.2 × 1.2 cm. Microscopic examination showed the same histological features as the first specimen (Fig. 2A and B) and a diagnosis of recurring EP of the gluteal region with no malignant transformation was verified.

## 3 Discussion

EP typically presents as soft-to-firm plaques or nodules. Most tumors are not associated with symptoms and have typically been indolent for an extended period before the patient seeks a physician for treatment. If the EP becomes infected and painful, the patients may present with bleeding or ulceration, which may lead to a wrong diagnosis at the first presentation [7]. The clinical presentation, in our case, was a suppurating lesion, which may have developed due to recurrent trauma to this particular site in the buttock, what had predisposed it to recurrent infection by various types of bacterial infection during the first and second episodes. To our knowledge, the gluteus as the location of EP was only reported twice before; in 2009, in a 60-year-old male by Sarma et al. followed by another case published in 2011, with typical histological observations in the buttock region of a 74-year-old man [8,9]. Although typically benign in nature, a small number of cases of recurrence of EP and its variants after incomplete excision have been reported [10,11]. Our case was relatively circumscribed and almost completely excised (with a least free surgical margin of 2 mm) and then recurred after 17 months giving a history of trauma which is not unexpected tale. Simple excision is the typical treatment for the deep lesion, however Superficial lesions may be treated with shave, electrosurgical destruction or simple excision [5]. Open debridement and biopsy for management of gluteal abscess are warranted in several cases, if the diagnosis is still unclear then adequate tissue has to be obtained [12].

We recommend simple excision with adequate margin in case of the mass lesion suspicious for EP in the gluteal region to ensure limitation of recurrence or malignant transformation to carcinoma. Eccrine porocarcinoma is a rare but aggressive type of skin tumor; it may arise denovo or complicate EP [13].

Histopathological examination is critical to establishing the diagnosis of various rare and recurrent neoplastic lesions including EP due to existence of various clinical and histological differential diagnoses, including benign and malignant lesions, such trichoepithelioma, basal cell carcinoma, hidradenoma papilliferum, follicular (inclusion) cysts, and eccrine porocarcinoma [[14], [15], [16]].

## 4 Conclusion

Gluteal region skin is an unusual location for a benign poroma. Early recognition and appropriate treatment at the initial presentation by complete resection with surgical margins of the EP and with histological confirmation and follow-up are crucial to ruling out and limitation of other diseases, such as recurrent lesions of malignant transformation.

## Declaration of Competing Interest

None declared by all the authors.

## Funding

No any funding sources for this article.

## Ethical approval

Local Research Ethics Committee approval was done.

## Consent

Written informed consent was obtained from the patient for publication of this case report

## Author contribution

-Abdulkarim Hasan: Histopathology interpretation, study design, work administration, participation in writing and final revision.

-Khalid Nafie: Lab data collection, imaging edition, abstract preparation and final revision.

-Khaled Monazea: Performing the procedure, study concept and writing the paper.

-Alsayed Othman: Data collection, writing the paper and final revision.

-Abdoh Salem: Performing the procedure of the first operation and data collection.

-Amal Ismail: writing the paper, Images editing, proofreading and final revision.

## Registration of research studies

NA (No human participants).

## Guarantor

Abdulkarim Hasan.

## Provenance and peer review

Not commissioned, externally peer-reviewed.
